# Micro-Erythrocyte Sedimentation Rate in Neonatal Sepsis of a Tertiary Hospital: A Descriptive Cross-sectional Study

**DOI:** 10.31729/jnma.4984

**Published:** 2020-06

**Authors:** Sunil Raja Manandhar, Rydam Basnet

**Affiliations:** 1 Neonatal Unit, Department of Pediatrics, Kathmandu Medical College Teaching Hospital, Sinamangal, Kathmandu, Nepal.

**Keywords:** *c-reactive protein*, *erythrocyte sedimentation rate*, *neonatal sepsis*

## Abstract

**Introduction:**

Neonatal sepsis is the most important cause of morbidity and mortality among low birth weight and preterm babies in developing countries. The main objective of this study is to find the level of micro-Erythrocyte sedimentation rate in neonatal sepsis.

**Methods:**

This is a descriptive cross-sectional study conducted at the neonatal unit over six months period (November 2019 to April 2020). All preterm, term and post-term babies with neonatal sepsis delivered at Kathmandu Medical College Teaching Hospital were enrolled. Ethical clearance was received from the Institutional Review Committee of Kathmandu Medical College (Ref: 181020191). Convenient sampling method was applied and statistical analysis was done with Statistical package for social sciences 19 version.

**Results:**

Out of 75 babies, confirm sepsis is 13 (17.3%), probable sepsis is 40 (53.4%) and suspected sepsis is 22 (29.2%). Micro-Erythrocyte sedimentation level is elevated (>15mm in 1^st^ hr) in 25 (33.3%) babies with a mean micro-Erythrocyte sedimentation level 9.32±5.4 (2-18) mm in 1^st^hr. The elevated micro- Erythrocyte sedimentation level was seen in relation to sepsis types and C-reactive protein.

**Conclusions:**

The bedside micro-Erythrocyte sedimentation level aids in the diagnosis of neonatal sepsis.

## INTRODUCTION

Neonatal sepsis is the most important cause of mortality among low birth weight and preterm babies in developing countries.^1^ In Nepal, the neonatal mortality rate is still high (21 per 1000 live births). Causes of neonatal deaths are sepsis, perinatal asphyxia, prematurity, and low birth weight.^2,3^ Maternal risk factors eg. Prolonged rupture of membranes (PROM) ≥ 18 hrs., positive high vaginal swab culture, intrapartum fever, and neonatal risk factors eg. birth weight <1500gm, gestational age <34 wks, low APGAR score contributes for sepsis.^4^

Sepsis is defne as signs and symptoms of infections with or without accompanying bacteremia with the growth of bacteria within frst month of life and consists of septicemia, meningitis, pneumonia, and urinary tract infection.^5^ Prompt diagnosis, good nursing care, and antibiotics aids to save babies with sepsis.^6^ Blood culture is the gold standard for sepsis diagnosis, but it is time-consuming and needs equipped lab.^7,8^

So, the main objective is to fnd out the micro-ESR level in neonatal sepsis at a tertiary hospital.

## METHODS

A descriptive cross-sectional study was carried out on neonates with neonatal sepsis at 10 bedded Neonatal Intensive Care Unit (NICU) of the Pediatrics Department of Kathmandu Medical College Teaching Hospital over six months period (November 2019 - April 2020). Perinatal Mortality Rate (PMR) of this tertiary hospital is 10 per 1000 births and Neonatal mortality rate (NMR) is 4.5 per 1000 live births.^9^

All preterm, term and post-term neonates admitted in the NICU with a diagnosis of suspected sepsis, probable sepsis and confrm sepsis were enrolled. Risk factors and laboratory criteria for the diagnosis of neonatal sepsis is mentioned in Box 1.^10^

Box. 1 **Risk factors, clinical features and laboratory criteria for neonatal sepsis.^10^**

- Maternal Risk Factors^1^
  - History of PROM for ≥ 18 hours
  - Foul smelling liquor
  - Spontaneous preterm labor < 37 weeks
  - Intrapartum fever ≥ 38^0^ C
- Neonatal Risk Factors^1,4,6^
  - Prematurity ≤ 34 wks
  - Birth weight ≤ 1.5 kg
  - Low Apgar score
  - Respiratory distress (tachypnea, grunting, increase oxygen requirement)
  - Apnea/cyanosis
  - Unstable temperature–hyperthermia (Fever) / hypothermia
  - Cardiovascular disturbance (tachycardia / bradycardia, poor peripheral perfusion)
  - Poor feeding
  - Lethargy, irritability and seizures
- Laboratory diagnostic criteria for neonatal sepsis^6^
  I) Direct method: Isolation of microorganisms in blood or urine or CSF culture.
  II) Indirect method: Septic screen consider to be positive if any two or more out of following fve parameters are present:
    - Leucopenia (TLC < 5000 /mm3)
    - Neutropenia (ANC < 1800/mm3)
    - Immature neutrophils to total neutrophils (I/T) ratio: > 0.2
    - Micro ESR: ≥ 15mm in 1st hour
    - C-Reactive Protein (CRP): positive

Suspected sepsis is defne as neonates who fulfll the following minimum three signs and symptoms eg. sclerema, lethargy, apnea, hypotonia, poor cry, poor feeding, respiratory distress, grunting, vomiting, fever, mottling of the skin and irritability with normal fve laboratory septic screening test mentioned in Box 1.^4,10^ A neonate clinically have above mentioned clinical signs and symptoms with at least two of the fve laboratory screening tests positive mentioned above in box 1 with negative blood culture is diagnosed as probable sepsis.^11^ Confrm sepsis is defne as neonate with above mentioned clinical sign/symptoms with positive two out of the fve laboratory screening tests mentioned above with having blood, urine or cerebrospinal fuid culture yielding an organism.^11^

Neonates fulflling inclusion criteria were drawn venous blood for blood culture and sensitivity (CS), total leukocyte count (TLC), diferential count (DC), absolute neutrophil count (ANC), hemoglobin(Hb), micro-erythrocyte sedimentation rate (micro-ESR), C- reactive protein (CRP) and peripheral smear for band cells. Blood parameters TLC/ DC/ Hb were performed by the coulter method. In a suspected case of pneumonia, a chest x-ray is done and lumbar puncture (LP) is done in suspected case of meningitis. Blood CS was done via a BACTEC 9050 automation system, Becton Dickinson, Ireland. In which 3ml venous blood was inoculated into BACTEC Ped Plus culture vial under complete aseptic conditions. Then blood was kept in the BACTEC 9050 blood culture instrument within 2 hr of collection and subcultures were done in positive cases to identify the causative organism according to the standard methods. Serum CRP level was measured by the semi-quantitative latex agglutination test (AVITEX CRP kits; Catalog No. OD023; supplied by Omega Diagnostics, UK). Urine routine and CS were sent in case of suspected urinary tract infection (UTI) by supra pubic aspiration. All the samples were sent to the laboratory within a half-hour of the procedure.

The following procedure was done for the estimation of micro-ESR at bedside Resuscitaire of newborns in the NICU. Blood was collected in a pre-heparinized micro hematocrit tube of 75 mm length with an internal diameter of 1.1mm and an external diameter of 1.5mm by heel prick technique. Air is not allowed to interrupt the column of blood to avoid false normal result and one end was sealed using clay wax. The micro hematocrit tube was then fxed vertically on a clay tray near the bedside with the identifcation of patient and the time of blood collection noted and left undisturbed for one hour. There after the distance from the highest point of the plasma column to the meniscus of the packed red cell column (height of the plasma column) of each tube was measured with a ruler after one hour. micro-ESR level was said to be elevated if the height of the plasma column measured were greater than 15mm/hr for all neonates irrespective of age.^12,13^ The clinical details and results of laboratory investigations were recorded in a pre-designed proforma. The management of neonatal sepsis was done as per the neonatal unit protocol. Babies with a lethal congenital malformation (eg. Meningomyelocel, Anencephaly, Gastroschisis,

Diaphragmatic Hernia) and Syndromic babies were excluded. Ethical clearance was received from the Institutional Review Committee (IRC) of Kathmandu Medical College (Ref: 181020191) and written consent was taken from the parents and possible complications of neonatal sepsis were explained. The neonates were evaluated by a thorough history from mother, maternal parameters at birth, and detail clinical examinations. Gestational age assessment was done by using modifed new Ballard score ^14^ and maternal and neonatal risk factors were assessed as mentioned in Box 1. Data were analyzed in Statistical package for social sciences (SPSS 19) version in the form of frequency, tables along with mean and standard deviation. Relation of Micro ESR with respect to types of sepsis and CRP were analyzed. The sample size was calculated as follows;

Sample size (n) = Z^2^ × (p × q)/e^2^

     = (1.96)^2^ × 0.05 × (1 – 0.05)/0.05)^2^

     = 3.84 × 0.05 × 0.95/0.0025

     = 0.1824/0.0025

     = 72.96

     n = 75 neonates

Where,

n = Sample size
Z = 1.96 at 95% Confdence Interval
p = Prevalence of Neonatal sepsis in previous
study = 5 % (Gomez B et al)^15^
q = 1- p
e = Margin of error, 5%

A total of 75 neonates were enrolled and a convenient sampling method was applied.

## RESULTS

A total of 2160 babies were delivered at KMCTH over six months period and 75 babies full flling the inclusion criteria were enrolled in this study. Among them, 45 (60%) babies were male and most of them were term 46 (61.3%) babies with 50 (66.7%) delivered by primi mothers. The mean birth weight observed was 2611±79 gms and the mean gestational age was 36.35±3.2 weeks. While analyzing clinical parameters, mean respiratory rate of enrolled babies was 76.63 ±18.7 breaths /min (20 -110) and mean heart rate was 162.71±23.6 beats /min (82- 200). During treatment in NICU, due to respiratory problem, 63 (84%) babies required Bubble CPAP ventilation with mean duration 58.25±33.4 hrs. (12-160) and 20 (26.6%) babies required mechanical ventilation with mean duration 35.55±18.7 hrs. (4-72). The mean maternal age was 26.52±3.6 yrs. (19–39) and 33 (44%) mothers had history of PROM with a mean duration 43.97±56.5 hrs. (18 -288). Similarly, the mean age of babies admitted in NICU was 3.99±4.8 days and mean hospital stay for 70 babies were 8.5±4.8 days and fve babies were expired during treatment of sepsis. (Table1).

**Table 1:** Neonatal and maternal characteristics (n = 75).

Variables		
Neonatal Characteristics	Mean	Range
Gestational Age	36.35±3.2	(24-40)wks.
Birth weight	2611±79	(600-4200)gms
Apgar Score at 1 min (45 babies)	7.05±1.6	(1-8)
Apgar Score at 5 min (45 babies)	8.23±1.3	(3-9)
Age of baby during NICU admission	3.99±4.8	(1^st^ day-22^nd^ days)
Clinical parameter of newborns	Mean	Range
Respiratory rate	76.63±18.7	(20-110) breaths/min
Heart rate	162.71±23.6	(82-200 ) beats/min
Bubble CPAP duration (63 babies)	58.25±33.4	(12-160)hrs
Mechanical ventilation duration(20 babies)	35.55±18.7	(4-72) hrs
Antibiotics use duration (75 babies)	8.1± 4	(3-21)days
Hospital Stay (70 babies)	8.5±4.8	(3-28)days
Maternal Characteristics	Mean	Range
Maternal Age	26.52±3.6	(19-39)yrs.
Prolonged rupture of membrane (PROM) (33 mothers)	43.97±56.5	(18-288 )hrs

Analyzing the maternal and neonatal risk factors for sepsis, in 33 (44%) babies, PROM was the commonest maternal risk factor whereas prematurity 22 (29.3%) followed by fast breathing 19 (25.3%) were the commonest neonatal risk factors (Table 2).

**Table 2:** Risk factors for sepsis (n = 75).

Maternal	n (%)
PROM	33 (44.0)
No risk	23 (30.6)
Maternal fever	5 (6.7)
Antepartum hemorrhage (APH)	3 (4.0)
Preeclampsia	3 (4.0)
Maternal Gestational diabetes	2 (2.7)
Maternal hypothyroidism	2 (2.7)
Eclampsia	1 (1.3)
Foul-smelling liquor	2 (2.7)
Rh Iso- immunization	1 (1.3)
Total	75 (100)
Neonatal risk factors	n (%)
Preterm	22 (29.3)
Fast breathing	19 (25.3)
Perinatal Asphyxia	7 (9.3)
Fever	7 (9.3)
Lethargy	7 (9.3)
LBW (Low Birth Weight)	5 (6.7)
Meconium aspiration	5 (6.7)
ELBW (Extremely LBW)	2 (2.7)
Big Baby	1(1.4)
Total	75 (100)

**Figure 1:**
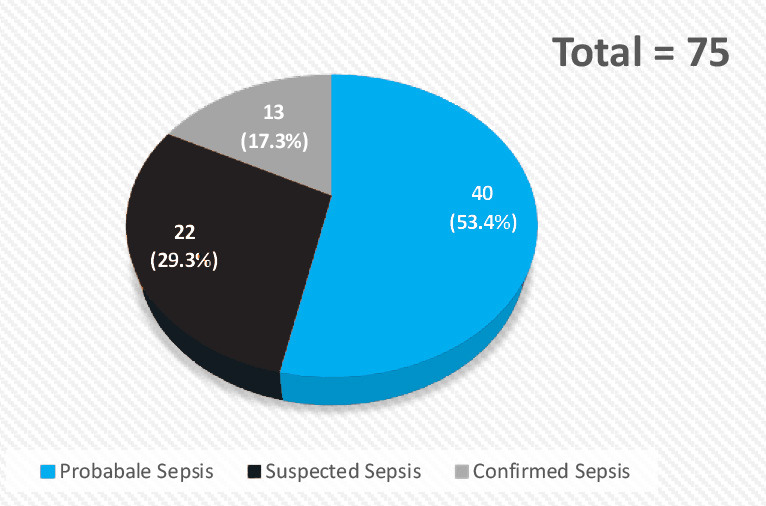
Pie Chart showing types of Neonatal Sepsis.

**Figure 2:**
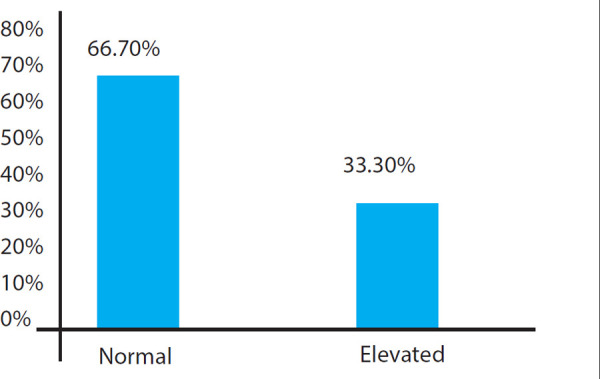
Bar diagram showing Micro-ESR level.

In this study, out of 75 babies, confrmed sepsis is 13 (17.3%), probable sepsis is 40 (53.4%) and suspected sepsis is 22 (29.3%) (Figure.1). The micro-ESR level is normal (≤ 15 mm in 1^st^ hr) in 50 (66.7%) babies and elevated (> 15mm in 1^st^ hr) in 25 (33.3%) babies. The mean micro-ESR level observed in this study is 9.32 ±5.4 (2-18) mm in 1^st^ hr. (Figure 2).

**Table:** 

	Mean	Range
M i r c o -ESR Level	9.32±5.4	(2-18) mm/1^st^ hr

Among 50 babies with normal micro-ESR level, one (2%) baby was diagnosed as confrm sepsis, 27 (54%) as probable sepsis and 22 (44%) babies as suspected sepsis. Whereas out of 25 babies with elevated micro- ESR level, 12 (48%) babies were diagnosed as confrm sepsis and 13 (52%) as probable sepsis. Similarly, among 50 babies with normal micro-ESR level, CRP was positive in 26 (52%) babies and negative in 24 (48%). Whereas out of 25 babies with elevated micro-ESR level, all 25 (100%) babies had positive CRP (Table 3).

**Table 3:** Micro–ESR level in accordance with sepsis types and CRP.

	Micro E SR Level	
Variables	Normal ≤15mm in 1 ^st^hr	Elevated >15mm in 1^st^hr
Sepsis Types	n (%)	n (%)
Confirm Sepsis	1 (2)	12 (48)
Probable Sepsis	27 (54)	13 (52)
Suspected Sepsis	22 (44)	0 (0)
Total	50 (100)	25 (100)
C-Reactive Protein (CRP)		
Negative	24 (48)	0 (0)
Positive	26 (52)	25 (100)
Total	50 (100)	25 (100)

## DISCUSSION

Neonatal Sepsis is the commonest cause of neonatal mortality and morbidity resulting in 30-50% of total neonatal deaths in developing countries.^16,17^ Considering its high mortality and morbidity, a specifc diagnostic marker eg. Interleukin 6, Interleukin 8, Procalcitonin with high sensitivity and specifcity is desirable in neonatal sepsis. However, these tests are not easily available in our country and also not cost-efective.^18^ Apart from other septic screening tests mentioned in Box 1, micro ESR is a single, cheap, easy to perform, less time consuming with sensitivity and specifcity of 63.3 % and 60% respectively.^19^

A study done by Kafe R et al. in Universal College of Medical Sciences, Bhairahawa, Nepal found 6% of babies with confrm sepsis and 12% had elevated micro –ESR level.^18^ Similarly, a study done by Ghaliyah AZ et al in Yenepoya Medical College Teaching Hospital, Mangalore, India found 32% confrm sepsis with a 38% elevated micro-ESR level.^4^ In our study also 13 (17.3%) babies had confrmed sepsis and a total of 25 (33.3%) babies had raised micro-ESR level showing the signifcance of micro-ESR level concerning confrm sepsis.

Shah GS et al. at BPKIHS, Dharan, Nepal described the maternal history of PROM, maternal history of foul-smelling liquor, prematurity, low birth weight and low Apgar score at birth were the strong risk factors for early-onset neonatal sepsis.^1^ Similarly, in this study, also maternal PROM (44%) and preterm (29.3%) were the commonest risk factors highlighting its major role for sepsis in babies.

CRP is an infammatory acute-phase reactant that promotes the healing of the injured tissue.^20^ CRP is also a good marker of sepsis with sensitivity and specifcity of 77.8% and 66.7% respectively.^21^ In Ghaliyah AZ et al study,^4^ out of 19 babies with elevated micro-ESR level, 12 (63.2%) has positive CRP whereas in our study all twenty-fve (100%) babies with elevated micro-ESR had positive CRP showing the signifcance of CRP with the raised micro-ESR level in sepsis diagnosis.

## CONCLUSIONS

Neonatal Sepsis is the commonest cause of neonatal mortality and morbidity.^15^ The bedside micro-ESR level test showed signifcance in the diagnosis of neonatal sepsis for better management in the NICU. Since this is a study of a single institution with convenient sampling, the outcome cannot be generalized.
